# Radiomics Features from Positron Emission Tomography with [^18^F] Fluorodeoxyglucose Can Help Predict Cervical Nodal Status in Patients with Head and Neck Cancer

**DOI:** 10.3390/cancers16223759

**Published:** 2024-11-07

**Authors:** Francesco Bianconi, Roberto Salis, Mario Luca Fravolini, Muhammad Usama Khan, Luca Filippi, Andrea Marongiu, Susanna Nuvoli, Angela Spanu, Barbara Palumbo

**Affiliations:** 1 Department of Engineering, Università degli Studi di Perugia, Via Goffredo Duranti 93, 06125 Perugia, Italy; mario.fravolini@unipg.it (M.L.F.); muhammadusama.khan@dottorandi.unipg.it (M.U.K.); 2 Unit of Nuclear Medicine, Department of Medicine, Surgery and Pharmacy, University of Sassari, 07100 Sassari, Italy; robsalis87@gmail.com (R.S.); amarongiu2@uniss.it (A.M.); smfnuvoli@uniss.it (S.N.); aspanu@uniss.it (A.S.); 3 Perugia Research Unit, CNIT—National Inter-University Consortium for Telecommunications, Via Goffredo Duranti 93, 06125 Perugia, Italy; 4 Department of Biomedicine and Prevention, University of Rome ‘Tor Vergata’, Via Montpellier 1, 00133 Rome, Italy; luca.filippi@ptvonline.it; 5 Section of Nuclear Medicine and Health Physics, Department of Medicine and Surgery, Università degli Studi di Perugia, Piazza Lucio Severi 1, 06132 Perugia, Italy; barbara.palumbo@unipg.it

**Keywords:** head and neck cancer, positron emission tomography, cervical lymph nodes, radiomics

## Abstract

*Background:* Detecting pathological lymph nodes (LNs) is crucial for establishing the proper clinical approach in patients with head and neck cancer (HNC). Positron emission tomography with [^18^F] fluorodeoxyglucose (FDG PET) has high diagnostic value, although it can yield false positives since FDG-avid LNs can also occur from non-cancerous diseases. *Objectives:* To explore if radiomics features from FDG PET can enhance the identification of pathological lymph nodes in head and neck cancer. *Materials and methods:* This study was carried out on n=51 cervical lymph nodes (26 negative, 25 positive) from a cohort of n=27 subjects, and the standard of reference was fine needle aspiration cytology or excisional biopsy. An initial set of 54 IBSI-compliant radiomics features, which was subsequently reduced to 31 after redundancy elimination, was considered for the analysis. Mann–Whitney U tests were performed to compare each feature between positive and negative LNs. Classification models based on two sets of features, PETBase (SUVmax, MTV and TLG) and PETRad (radiomics features), respectively, were trained using logistic regression, support vector machines and Gaussian naïve Bayes, and their performance was compared. Accuracy was estimated via leave-one-out cross-validation. *Results:* We identified via univariate analysis 21 features that were statistically different between positive and negative LNs. In particular, dispersion features indicated that positive LNs had higher uptake non-uniformity than the negative ones. AUC, sensitivity, specificity and accuracy obtained with logistic regression were, respectively, 0.840, 68.0%, 89.5% and 80.4% for PETBase and 0.880, 72.0%, 90.0% and 82.4% for PETRad. The other classification models showed the same trend. *Conclusions:* Radiomics features from FDG PET can improve the diagnostic accuracy of LN status in HNC.

## Simple Summary

Detecting the lymph-node (LN) status plays a pivotal role in the management of patients with head and neck cancer (HNC). In this retrospective study we aimed to evaluate the possible benefits of using FDG PET radiomics to characterise the state of lymph nodes in HNC. Experimenting on a population of 51 lymph-nodes from 27 subjects we identified 21 radiomics features that were statistically different between the positive and negative lymph-node groups. Prediction models based on the radiomics features (PETRad) and semi-quantitative PET features (PETBase) were compared showing that PETRad provided better accuracy, sensitivity and specificity than the PETBase. We conclude that the use of radiomics features from FDG PET can improve the diagnostic accuracy of LN status in HNC.

## 1. Introduction

Head and neck cancer (HNC) is estimated to be the seventh most common type of cancer worldwide and the eighth leading cause of cancer-related fatalities at a global level [1,2]. In Italy, there were 9750 new cases and 3800 fatalities in 2022, with an estimated five-year survival rate of 59% for men and 62% for women [3].

Therapeutic options differ depending on the disease status when first detected, and include surgical excission, radiotherapy, chemotherapy or a combination of these (multimodal treatment). The presence of pathological lymph nodes (LNs), in particular, is crucial for patient staging and for determining the proper clinical approach, which should always aim at achieving the highest possible cure rate while minimising the risk of morbidity [4]. An assessment of LN status is first conducted at imaging using one or more modalities from the following: ultrasound (US), computed tomography (CT), magnetic resonance imaging (MRI) and positron emission tomography (PET). This is then followed by cytological and/or histological examination in suspicious cases. In this context, positron emission tomography/computed tomography with 18-F fluorodeoxyglucose (FDG PET in the remainder) plays a pivotal role in HNC tumour staging and in the evaluation of response to therapy [5,6]. There is evidence that FDG PET has a high diagnostic value for the identification of metastatic LNs as positive LNs are typically FDG-avid [6,7,8]; however, PET-positive LNs may also arise from benign conditions such as reactive lymphadenopathy [9].

In recent years, various studies have suggested that quantitative image features coupled with machine leaning methods (*radiomics*) can improve the prediction of LN status in a number of oncological disorders [10,11,12], including head and neck cancer [1,13,14]. Romeo et al. [15] demonstrated the ability of radiomics features from contrast-enhanced CT to predict tumour grade and nodal status in squamous-cell carcinoma of the oropharynx and oral cavity. Bardosi et. al [16] used the same method to distinguish between pathologic and non-pathologic LNs in head and neck squamous-cell carcinoma (HNSCC). In [17], Zheng et al. showed the utility of radiomics analysis on FDG PET/CT for differentiating lymphomatous vs. cancerous lymph nodes, whereas a study by Dohopolski et al. [18] supported the use of deep learning on fused PET/CT images to predict lymph node metastasis in oropharingeal squamous-cell carcinoma. Tsai et al. [19] demonstrated that the radiomics feature in CT can be useful to predict cervical lymph node recurrence in HNC patients undergoing definitive radiotherapy. Of late, van Staalduinen et al. [20] established the additional value of texture features from MRI in the differential diagnosis of indeterminate lymph nodes in patients with HNSCC.

Recent reviews [13,14,21] indicate that most radiomics studies investigating LN status in HNC are based on CT, MRI or fused PET/CT images, whereas PET alone has received less attention in comparison. This is not surprising if we consider that PET has a lower spatial resolution than CT or MRI and that lymph nodes are usually small, hence there are fewer voxels available for texture analysis in PET than in other imaging modalities. This discourages people from using high-order texture features, such as grey-level co-occurrence matrices, grey-level size-zone matrices and the like, for which a minimum of 64 voxels is usually recommended [22]. Instead, simpler radiomics features such as the morphological, intensity-based and histogram-based, appear more appropriate in this case. Furthermore, these features offer advantages in terms of interpretability, as they can be linked to intuitive concepts such as shape irregularity, uptake heterogeneity and the presence of hot-spots [23].

The objective of this study was to determine whether morphological and first-order texture features from FDG PET could help to improve the diagnostic process of suspicious LNs in head and neck cancer on FDG PET. In this respect, we performed a statistical and machine learning analysis on fifty-one IBSI-compliant radiomics features to determine whether they could improve the diagnostic accuracy achieved using semi-quantitative parameters alone—i.e., SUVmax, metabolic tumour volume (MTV) and total lesion glycolysis (TLG).

## 2. Materials and Methods

### 2.1. Patient Population

**Figure 1:**
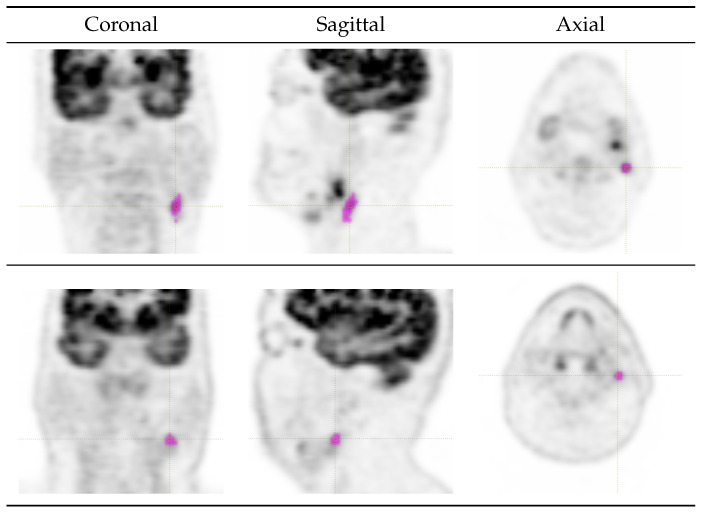
FDG PET images of two sample cases from the study population. (**Top row**) A positive left mid-jugular lymph node in a 71-year-old male (anonymous_id = f051, nodule_id = N_00; SUVmean = 3.62, SUVmax = 6.61). (**Bottom row**) A negative left upper-jugular lymph node in a 58-year-old male (anonymous_id = 6d32, nodule_id = N_01; SUVmean = 3.63, SUVmax = 4.42). Purple overlays indicate the manually generated ROIs. Further data are available in the data-spreadsheet.xlsx file provided in the Supplementary Materials (use the given anonymous IDs to identify patient and lymph node in the data table).

This study was carried out on n=51 cervical lymph nodes (26 negative, 25 positive) from a cohort of 27 subjects (male = 25, female = 26; age = 62.2 ± 6.3 [48–77 yr]) who received baseline FDG PET scans for clinical examination at the Unit of Nuclear Medicine of the Department of Medicine, Surgery and Pharmacy at the Università degli Studi di Sassari, Sassari, Italy, between March 2015 and September 2023. All of the patients were affected by histologically proven head and neck squamous-cell carcinomas (HNSCCs) with the primary lesions distributed as follows: oral cavity/oropharynx (n=10), laringopharynx (n=5), nasopharynx (n=1), multiple sites (n=1) and unknown/unspecified (n=10). The lymph node sites were as follows: upper jugular (n=22), mid jugular (n=14), lower jugular and medial supraclavicular (n=8), posterior triangle and supraclavicular (n=4), submental and submandibular (n=2) and anterior cervical (n=1). The standard of reference for lymph node status (positive or negative) was fine needle aspiration cytology (FNAC) or excisional biopsy. Two sample cases with ROI overlays are shown in Figure 1.

### 2.2. Image Acquisition

All the patients underwent FDG PET/CT examination with a Discovery 710 PET/CT scanner (GE HealthCare Technologies Inc., Chicago, IL, USA). In compliance with the AIMN protocol [24], the subjects were instructed to fast for at least 6 h before the procedure; then blood glucose tests were performed to make sure that the level was below 150 mg/dL. An initial low-dose (120–140 kVp) CT scan for attenuation correction and anatomical correlation was acquired in helicoidal mode, starting approximately 1 h after radiotracer injection. The CT images had the following characteristics: size 512 px × 512 px, in-plane pixel spacing 1.37 mm × 1.37 mm, slice thickness 3.75 mm and spacing between slices 3.27 mm. The PET signal was reconstructed by ordered subset expectation maximisation with point spread function recovery and time-of-flight (VPFXS) into images with the following characteristics: matrix size 256 px × 256 px, in-plane pixel spacing 2.73 mm × 2.73 mm and slice thickness 3.27 mm.

### 2.3. Lesion Delineation

Regions of interest (ROIs) corresponding to each lymph node were delineated manually on the fused PET/CT images by a nuclear medicine resident (R.S., >3 yr experience) under the supervision of a senior nuclear medicine specialist (B.P., >20 yr experience). Segmentation and feature extraction (see Section 2.4) were both performed via LIFEx v7.3.0 (LITO-Curie, SHFJ-CEA, CNRS, Univ. Paris Sud, University Paris Saclay, Orsay, France [25]).

### 2.4. Feature Extraction

A set of 54 IBSI-compliant [26,27] shape and texture features was initially included in this study. This comprised 12 morphological features, 19 first-order intensity-based features and 23 first-order histogram-based features.

There are three main reasons why we limited our study to these groups of features. The first is that the ROIs corresponding to the lesions investigated in this work (lymph nodes) are very small. As a consequence, we avoided the use of higher-order texture features, for which a minimum of 64 voxels is usually recommended (see also [22,28] on this point), and a significant fraction of our lesions is below that threshold. The second is that the evaluation of a large number of radiomics features on a small sample dataset can easily lead to false discovery [29]. Consequently, given the contained sample size of our population, we wanted to keep the number of features low. The third is that the features used here offer advantages in terms of interpretability, as they can be linked to intuitive concepts such as shape irregularity, uptake heterogeneity and presence of hot-spots.

For histogram-based features, we used absolute intensity rescaling between 0 and 20 SUV and quantisation into 64 bins (the same settings as in [17] and the default settings in the LIFEx version are used here). Apart from the above information, the images received no filtering, whereas other preprocessing steps such as spatial resampling were not required given the single-centre nature of the dataset (images acquired with the same scanner and settings).

#### Feature Selection

A subset of 31 features (respectively, six morphological, 18 first-order intensity-based and seven first-order histogram features—see Table 1 for the complete list) was retained from the initial 54 after eliminating redundancies. The process is summarised below:

(i) Some features are equivalent by definition (e.g., 50th intensity percentile and intensity median); in this case, we only retained one feature per group.
(ii) Some groups of morphological features can be obtained from one another by simple mathematical transformations, and therefore are strongly correlated [30]. Hence, for each of such groups, we retained just one feature. For instance, the morphological features compactness 1, compactness 2, sphericity and spherical disproportion (respectively, corresponding to IBSI codes [26,27]: SKGS, BQWJ, QCFX and KRCK) can be obtained from one another through mathematical transformations. We therefore kept only one feature of this group, i.e., sphericity.
(iii) Under fixed-bin-width absolute quantisation, first-order histogram-based features are a discretised approximation of their first-order intensity-based counterparts. Samples of such pairs of features are intensity-based skewness (KE2A) and histogram-based skewness (88K1), as well as intensity-based kurtosis (IPH6) and histogram-based kurtosis (C317). For all of these pairs, we only retained the intensity-based feature.

### 2.5. Statistical Analyses

#### 2.5.1. Univariate Analysis

Unpaired non-parametric Mann–Whitney U test was performed to compare each radiomics feature between the positive and negative lymph node groups. A Bonferroni-corrected *p*-Value ≤0.05 was considered statistically significant.

#### 2.5.2. Cut-Off Analysis

Optimal cut-off values for the statistically significant features (Table 1) were estimated by maximisation of the Youden index (*J*) [31]. Operatively, we generated, for each feature, n=50 candidate cut-off points by uniformly sampling the 10th–90th-percentile interval (to exclude tails) and selected the one that achieved the highest *J*.

#### 2.5.3. Machine Learning Analysis

Prediction models based on (a) logistic regression with $L_{2}$ penalty term and regularisation strength C=0.1, (b) support vector classifier with radial basis kernel and penalisation factor C=1.0 (SVM in the remainder) and (c) Gaussian naïve Bayes (Gaussian NB) were built upon two feature sets, respectively, denoted as PETBase and PETRad. PETBase was intended as a control set and included three standard PET quantitative parameters: metabolic tumour volume, SUVmax and total lesion glycolysis; whereas PETRad comprised all the features that tested positive for statistically significant differences between the two lymph node groups (see Table 1).

The predictive performance of PETBase and PETRad was estimated through leave-one-out cross-validation (LOOCV) protected for patient identity. That is, in each split, we took one lymph node at a time as the test set, and the remaining ones as the train set; furthermore, we removed from the train set all the lymph nodes belonging to the same subject as the lymph node in the test set. This way, we ensured that lymph nodes from the same patient could not be in the train and test set at the same time. The procedure was repeated for all the lymph nodes in the datasets; hence, we had as many splits as lymph nodes. The primary benefit of this estimation procedure is that it offers a comprehensive assessment of the model’s performance because each lymph node in the dataset is used for testing. Furthermore, it avoids potential bias related to an arbitrary split into the train and test sets. Area under curve (AUC), sensitivity, specificity and accuracy were the performance metrics. Confidence intervals for accuracy were also estimated, assuming a binomial distribution of the outcome (correct vs. wrong prediction) and standard approximation [32].

Given the difference in magnitude of the features involved—as clearly shown in Table 1—feature normalisation by zero-mean unit-variance (*z*-score) was also applied. To avoid data leakage, the data were always split into train and test sets first, then the scaler was preliminarily fit and applied to the train set, and eventually, the same normalisation parameters were used to scale the test set. All the analyses were carried out using Python v3.10.4 with functions from Pandas v1.4.2, SciPy 1.9.1, scikit-learn 1.0.2 and statsmodels 0.14.0.

## 3. Results

### 3.1. Univariate and Cut-Off Analysis

For the Mann–Whitney U test, twenty-one features showed statistically significant differences between positive and negative lymph nodes. Among them, we observed that measures of tendency like mean (SUVmean), max (SUVmax), median, energy and root mean square were higher in the positive group, as we would reasonably expect (Table 1 and Table 2).

Dispersion parameters such as variance, histogram entropy, range, interquartile range and absolute deviation (mean, robust mean and median) were also higher in the positive group, whereas uniformity was lower. This indicates that negative lymph nodes had a more uniform uptake distribution than the positive ones. Kurtosis was also significantly higher in the positive group, suggesting the presence of outliers (i.e., hot/cold spots) in the uptake distribution. Volume and surface area were also significantly higher in the positive group, which is reasonable.

### 3.2. Machine Learning Analysis

The accuracies of the PETBase and PETRad feature sets for the classification of the nodal status are reported in Table 3. We observe that with all the classification models (linear regression, SVC and Gaussian NB), the AUC, sensitivity, specificity and accuracy of PETRad were all higher than those achieved by the PETBase feature set.

## 4. Discussion

FDG PET has a well-established role in oncology, particularly for staging and monitoring therapeutic response. Even if MRI provides an excellent resolution for delineating primary tumours, and CT is widely used as a staging tool, FDG PET offers several unique advantages. As a whole-body imaging modality, it is highly effective in detecting distant metastases, in identifying synchronous second primaries, in locating carcinomas of unknown primary origin and in detecting residual or recurrent disease. For nodal staging, PET imaging is particularly valuable because it can reveal abnormal tracer uptake in pathological lymph nodes even before anatomical changes—such as enlargement or morphological abnormalities—occur. This offers superior sensitivity compared to CT or MRI for detecting nodal metastatic involvement.

In this study, we have investigated the potential utility of radiomics analysis on FDG PET for differentiating positive vs. negative LNs in patients with head and neck cancer, where determining nodal involvement plays a major role in the clinical management of this disease.

After performing univariate analysis, we found that positive LNs had higher uptake than the negative ones, which is in agreement with the current literature. Similar findings have been reported by Lim et al. [33] (mean SUVmax of negative and positive LNs, respectively, were 1.55 and 5.0), Koekkoek-Doll et al. [34] (median SUVmax of negative and positive LNs, respectively, were 4.3 and 11.0), and, more recently, by Bianchini et al. [35] (mean SUVmax of negative and positive LNs, respectively, were 6.51 and 16.69).

Interestingly, dispersion indices were also higher in the positive group, suggesting that uptake heterogeneity is an indicator of malignancy. Although these results are completely novel as far as it concerns cervical LNs evaluated at FDG PET, the link between uptake heterogeneity and malignancy/poor prognosis is consistent with that already established for primary lesions. For instance, in [36,37], it was found that the intra-tumoural heterogeneity of FDG uptake significantly correlates with tumour aggressiveness and is a predictor of survival in nasopharyngeal carcinoma. More generally, this result is in agreement with the evidence that uptake heterogeneity on PET correlates with malignancy/aggressiveness in solid tumours [38].

Machine learning analysis showed that radiomics features achieved better AUC, sensitivity, specificity and accuracy than basic quantitative parameters, therefore demonstrating the additional utility of radiomics features in predicting LN status. The analysis revealed moderate sensitivity and fairly good specificity, a trend consistent with that reported in a previous meta-analysis of non-radiomics studies [39]. However, we found that radiomics features could increase sensitivity by around 4.0 pp compared with semi-quantitative parameters. Altogether, the performance gain was contained; nonetheless, it remained consistent across the three classification models that were examined (Gaussian NB, SVM and logistic regression).

On the whole, our results indicate that machine learning applied to morphological and first-order texture features from FDG PET can improve the prediction of the nodal status in patients with head and neck cancer. The confidence intervals for accuracy (Table 3), however, show that there is an overlap between the PETBase and PETRad models, suggesting that the findings should be further confirmed in larger cohorts of subjects. Future studies, conducted on larger, ideally perspective and multi-centre cohorts of patients should also evaluate whether the gain is clinically significant to justify the use of radiomics features in clinical practice.

Although in recent years radiomics has catalysed significant interest within the scientific community, this discipline is still some way from immediate implementation in clinical practice. Integrating radiomics into the clinical workflow involves challenges such as computational requirements, specialized training and cost. Advanced infrastructure and dedicated software are essential for processing radiomics data, and clinicians would require targeted training in quantitative imaging interpretation. Initial costs may also be substantial, particularly in resource-limited settings. However, as radiomics software becomes more standardized and user-friendly, and as radiology training increasingly incorporates these skills, these barriers may progressively reduce. In this context, accumulating evidence of radiomics’ clinical value and cost-effectiveness could drive investment and encourage broader adoption in clinical settings.

## 5. Limitations and Future Work

This work is not exempt from limitations. In particular, it was based on a retrospective, single-centre patient population of relatively small size. It also relies on internal validation based on LOOCV, but lacks external validation. Future studies should aim to validate these preliminary results in larger, ideally perspective and multi-centre cohorts of patients to strengthen generalizability. Preprocessing operations like filtering were not investigated in the present communication; however, a systematic analysis of this could be carried out in the future on larger cohorts of patients. Likewise, feature selection beyond redundancy elimination and univariate analysis was not conducted here, but could be an interesting subject for future studies. Finally, the combination of radiomics features from different imaging modalities such as PET, CT, MRI and ultrasound could be inveatigated as a means to improve the prediction of lymph node status in patients with HNC.

## Figures and Tables

**Table 1:** Results of univariate analysis. Key to fields/symbols: Class = feature class. Name = feature name. P = positive group. N = negative group. Corr. $\bar{p}$-Value = Bonferroni-corrected *p*-Value. Boldface $\bar{p}$-Values denote statistically significant differences. Units of measure are reported in square brackets by each feature name; the symbol ‘*’ indicates dimensionless; ‘a.u.’ is arbitrary units.

Class	Name	Mean N	Mean P	$\bar{p}$-Value
MORPHOLOGICAL	Volume (= MTV) [mm^3^]	813.84	8509.69	**0.007**
MORPHOLOGICAL	SurfaceArea [mm^2^]	494.51	1970.33	**0.015**
MORPHOLOGICAL	Sphericity [*]	0.84	0.82	1.000
MORPHOLOGICAL	CentreOfMassShift [mm]	0.52	0.64	1.000
MORPHOLOGICAL	Maximum3DDiameter [mm]	16.85	27.56	0.093
MORPHOLOGICAL	IntegratedIntensity (=TLG) [mm^3^ × SUVbw]	2034.21	60,074.73	**0.002**
INTENSITY-BASED	Mean [SUVbw]	2.34	4.78	**0.001**
INTENSITY-BASED	Variance [SUVbw]	0.37	3.16	**0.001**
INTENSITY-BASED	Skewness [*]	0.11	0.42	0.251
INTENSITY-BASED	Kurtosis [*]	−0.78	−0.19	**0.002**
INTENSITY-BASED	Median [SUVbw]	2.32	4.62	**0.001**
INTENSITY-BASED	MinimumGreyLevel (=SUVmin) [SUVbw]	1.44	2.07	1.000
INTENSITY-BASED	10thPercentile [SUVbw]	1.69	2.96	**0.013**
INTENSITY-BASED	90thPercentile [SUVbw]	3.04	6.93	**0.001**
INTENSITY-BASED	MaximumGreyLevel (=SUVmax) [SUVbw]	3.37	8.98	**0.000**
INTENSITY-BASED	InterquartileRange [SUVbw]	0.74	2.14	**0.002**
INTENSITY-BASED	Range [SUVbw]	1.93	6.91	**0.001**
INTENSITY-BASED	MeanAbsoluteDeviation [SUVbw]	0.42	1.24	**0.001**
INTENSITY-BASED	RobustMeanAbsoluteDeviation [SUVbw]	0.33	0.91	**0.002**
INTENSITY-BASED	MedianAbsoluteDeviation [SUVbw]	0.41	1.22	**0.001**
INTENSITY-BASED	CoefficientOfVariation [*]	0.21	0.31	0.201
INTENSITY-BASED	QuartileCoefficientOfDispersion [*]	0.16	0.23	0.479
INTENSITY-BASED	Energy [SUVbw^2^]	280.85	20,566.06	**0.001**
INTENSITY-BASED	RootMeanSquare [SUVbw]	2.41	5.05	**0.001**
INTENSITY-HISTOGRAM	IntensityHistogramMode [a.u.]	7.54	14.20	**0.004**
INTENSITY-HISTOGRAM	IntensityHistogramEntropyLog2 [bits]	2.37	3.68	**0.001**
INTENSITY-HISTOGRAM	Uniformity [*]	0.24	0.12	**0.002**
INTENSITY-HISTOGRAM	MaximumHistogramGradient [a.u.]	4.06	7.52	1.000
INTENSITY-HISTOGRAM	MaximumHistogramGradientGreyLevel [a.u.]	5.81	10.60	0.085
INTENSITY-HISTOGRAM	MinimumHistogramGradient [a.u.]	−3.94	−7.04	1.000
INTENSITY-HISTOGRAM	MinimumHistogramGradientGreyLevel [a.u.]	9.19	17.04	**0.001**

**Table 2:** Results of cut-off analysis. Key to fields/symbols: Class = feature class. Name = feature name. Cut-off = optimal threshold value for malignancy. See Table 1 for the units of measure.

Class	Name	Cut-Off
MORPHOLOGICAL	Volume	>496.76
MORPHOLOGICAL	SurfaceArea	>362.38
MORPHOLOGICAL	IntegratedIntensity	>1100.02
INTENSITY-BASED	Mean	>2.82
INTENSITY-BASED	Variance	>0.72
INTENSITY-BASED	Kurtosis	>−0.91
INTENSITY-BASED	Median	>3.00
INTENSITY-BASED	10thPercentile	>1.61
INTENSITY-BASED	90thPercentile	>3.91
INTENSITY-BASED	MaximumGreyLevel	>4.56
INTENSITY-BASED	InterquartileRange	>1.45
INTENSITY-BASED	Range	>3.59
INTENSITY-BASED	MeanAbsoluteDeviation	>0.71
INTENSITY-BASED	RobustMeanAbsoluteDeviation	>0.56
INTENSITY-BASED	MedianAbsoluteDeviation	>0.71
INTENSITY-BASED	Energy	>52.52
INTENSITY-BASED	RootMeanSquare	>2.80
INTENSITY-HISTOGRAM	IntensityHistogramMode	>9.16
INTENSITY-HISTOGRAM	IntensityHistogramEntropyLog2	>3.23
INTENSITY-HISTOGRAM	Uniformity	<0.11
INTENSITY-HISTOGRAM	MinimumHistogramGradientGreyLevel	>11.20

**Table 3:** Results of machine learning analysis—prediction accuracy (positive vs. negative lymph nodes). Key to fields/symbols: Feature set = set of radiomics features; Classifier = classification model; AUC = area under curve; Sens. = sensitivity; Spec. = specificity; Acc. = accuracy; Acc. CI = estimated 95% confidence interval for accuracy.

Feature Set	Classifier	AUC	Sens.	Spec.	Acc.	Acc. CI
PETBase	Logistic regression	0.840	68.0%	89.5%	80.4%	69.5–91.3%
PETRad		0.880	72.0%	90.0%	82.4%	71.9–92.8%
PETBase	SVM	0.811	72.0%	90.0%	82.4%	71.9–92.8%
PETRad		0.823	76.0%	90.5%	84.3%	74.3–94.3%
PETBase	Gaussian NB	0.798	56.0%	87.5%	74.5%	62.5–86.5%
PETRad		0.846	64.0%	88.9%	78.4%	67.1–89.7%

## Data Availability

Anonymised patients’ metadata and radiomics features are provided as Supplementary Materials in the data-spreadsheet.xlsx file. Original FDG PET scans are not available due to privacy restrictions.

## Supplementary Materials

The following supporting information can be downloaded at: https://www.mdpi.com/article/10.3390/cancers16223759/s1. Anonymised patients’ metadata and radiomics features (data-spreadsheet.xlsx).

## Abbreviations

The following abbreviations are used in this manuscript:

AIMN	Associazione italiana di medicina nucleare, imaging molecolare e terapia
AUC	Area under the curve
CI	Confidence interval
CT	Computed tomography
FDG PET	Positron emission tomography/computed tomography with [^18^F] fluorodeoxyglucose
FNAC	Fine needle aspiration cytology
HNC	Head and neck cancer
HNSCC	Head and neck squamous-cell carcinoma
IBSI	Image biomarker standardisation initiative
LN	Lymph node(s)
LOOCV	Leave-one-out cross-validation
MRI	Magnetic resonance imaging
MTV	Metabolic tumour volume
ROI	Region(s) of interest
SVM	Support vector machine(s)
SUV	Standardised uptake value
TLG	Total lesion glycolysis
US	Ultrasound
